# A systematic review and meta-analysis, investigating dose and time of fluvoxamine treatment efficacy for COVID-19 clinical deterioration, death, and Long-COVID complications

**DOI:** 10.1038/s41598-024-64260-9

**Published:** 2024-06-12

**Authors:** Mani Iyer Prasanth, Dhammika Leshan Wannigama, Angela Michelle Reiersen, Premrutai Thitilertdecha, Anchalee Prasansuklab, Tewin Tencomnao, Sirikalaya Brimson, James Michael Brimson

**Affiliations:** 1 Natural Products for Neuroprotection and Anti-Ageing (Neur-Age Natura) Research Unit, Chulalongkorn University, Bangkok, 10330 Thailand; 2 Department of Clinical Chemistry, Faculty of Allied Health Sciences, Chulalongkorn University, Bangkok, Thailand; 3 Department of Infectious Diseases and Infection Control, Yamagata Prefectural Central Hospital, Yamagata, Japan; 4 Department of Microbiology, Faculty of Medicine, King Chulalongkorn Memorial Hospital, Chulalongkorn University, Thai Red Cross Society, Bangkok, Thailand; 5 Yamagata Prefectural University of Health Sciences, Kamiyanagi, Yamagata, 990-2212 Japan; 6 Pathogen Hunter’s Research Collaborative Team, Department of Infectious Diseases and Infection Control, Yamagata Prefectural Central Hospital, Yamagata, Japan; 7 Department of Psychiatry, School of Medicine, Washington University in St. Louis, St. Louis, MO USA; 8 Siriraj Research Group in Immunobiology and Therapeutic Sciences, Faculty of Medicine Siriraj Hospital, Mahidol University, Bangkok, Thailand; 9 College of Public Health Sciences, Chulalongkorn University, Bangkok, Thailand; 10 Department of Clinical Microscopy, Faculty of Allied Health Sciences, Chulalongkorn University, Bangkok, Thailand; 11 Research, Innovation and International Affairs, Faculty of Allied Health Sciences, Chulalongkorn University, 154 Rama 1 Road, Pathumwan, Wang Mai, Bangkok, 10330 Thailand

**Keywords:** (E)‐5‐methoxy‐1‐[4‐(trifluoromethyl)phenyl]pentan‐1‐one O‐2‐aminoethyl oxime), SARS-CoV-2, Pandemic, Sigma-1 receptor (σ1R), Drug repurposing, Pandemic, Coronavirus, Antidepressant, Neurochemistry, Disease prevention, Public health, Therapeutics, Viral infection, Drug safety, Pharmacology, Target identification, Neurological disorders, Infectious diseases, Neurological disorders, Psychiatric disorders, Respiratory tract diseases

## Abstract

There have been 774,075,242 cases of COVID-19 and 7,012,986 deaths worldwide as of January 2024. In the early stages of the pandemic, there was an urgent need to reduce the severity of the disease and prevent the need for hospitalization to avoid stress on healthcare systems worldwide. The repurposing of drugs to prevent clinical deterioration of COVID-19 patients was trialed in many studies using many different drugs. Fluvoxamine (an SSRI and sigma-1 receptor agonist) was initially identified to potentially provide beneficial effects in COVID-19-infected patients, preventing clinical deterioration and the need for hospitalization. Fourteen clinical studies have been carried out to date, with seven of those being randomized placebo-controlled studies. This systematic review and meta-analysis covers the literature from the outbreak of SARS-CoV-2 in late 2019 until January 2024. Search terms related to fluvoxamine, such as its trade names and chemical names, along with words related to COVID-19, such as SARS-CoV-2 and coronavirus, were used in literature databases including PubMed, Google Scholar, Scopus, and the ClinicalTrials.gov database from NIH, to identify the trials used in the subsequent analysis. Clinical deterioration and death data were extracted from these studies where available and used in the meta-analysis. A total of 7153 patients were studied across 14 studies (both open-label and double-blind placebo-controlled). 681 out of 3553 (19.17%) in the standard care group and 255 out of 3600 (7.08%) in the fluvoxamine-treated group experienced clinical deterioration. The estimated average log odds ratio was 1.087 (95% CI 0.200 to 1.973), which differed significantly from zero (z = 2.402, *p* = 0.016). The seven placebo-controlled studies resulted in a log odds ratio of 0.359 (95% CI 0.1111 to 0.5294), which differed significantly from zero (z = 3.103, *p* = 0.002). The results of this study identified fluvoxamine as effective in preventing clinical deterioration, and subgrouping analysis suggests that earlier treatment with a dose of 200 mg or above provides the best outcomes. We hope the outcomes of this study can help design future studies into respiratory viral infections and potentially improve clinical outcomes.

## Introduction

The outbreak of COVID-19 was caused by the severe acute respiratory syndrome coronavirus 2 (SARS-CoV-2)^1^. Since its outbreak in Wuhan, China, in December 2019, COVID-19 has rapidly spread worldwide, leading to one of the deadliest pandemics in modern history^2,3^. With over 774 million cases and 7 million deaths globally by January 2024, the virus has had a profound impact on public health^4^. Various mutations, such as the delta, lambda, mu, and omicron variants, have continued to drive the death toll and have led to widespread lockdowns to control the spread of the virus^5–7^. However, the advent of vaccines has significantly reduced the disease's severity and mortality rate.

COVID-19 is now considered in the differential diagnosis for several common neurological syndromes, including encephalopathy, encephalitis, stroke, and Guillain-Barré syndrome^8^. Approximately half of COVID-19 survivors experience long-term effects, known as post-acute sequelae of COVID-19 (PASC) or Long-covid^9^. Symptoms of Long-covid encompass a wide range of issues, including sleep disturbances, anxiety, depression, brain fog, and fatigue. These persistent symptoms are linked to several mechanisms, such as neuroinflammation, blood–brain barrier breakdown, and neurodegeneration^10^.

One drug that could be used for treatment that emerged early in the pandemic is fluvoxamine, which was initially used for obsessive–compulsive disorder and major depression^11^. Fluvoxamine was tested for the treatment of COVID-19 due to its sigma-1 receptor (σ1R) agonist action, which has anti-inflammatory effects^12^. This action helps prevent excessive cytokine production, potentially reducing clinical deterioration in COVID-19 patients. Additional hypothesized mechanisms include inhibition of acid sphingomyelinase and platelet and mast cell inhibition. Research has shown that fluvoxamine can significantly reduce SARS-CoV-2 replication, indicating its potential effectiveness in treating COVID-19^13^.

The σ1R is crucial in the early stages of viral RNA replication^14,15^. Studies have demonstrated that knocking out the SIGMAR1 gene reduces SARS-CoV-2 replication^16^. Fluvoxamine promotes σ1R dissociation from binding immunoglobulin protein (BiP)/GRP78, enhancing chaperone activity^13^. Previous studies have confirmed that fluvoxamine binds to σ1R in the human brain at therapeutic doses, highlighting its potential as a COVID-19 treatment^17^.

Long-covid includes persistent symptoms that can last weeks or months after the acute infection phase^18,19^. These symptoms range from fatigue and shortness of breath to chest pain, joint pain, and cognitive difficulties, often called "brain fog." Other reported symptoms include persistent loss of taste or smell, headaches, heart palpitations, and sleep disturbances. The severity and duration of these symptoms vary widely among individuals. There is growing evidence that SARS-CoV-2 can breach the blood–brain barrier, leading to psychiatric and neurological symptoms in COVID-19 survivors. It is believed that a combination of viral persistence, immune system dysregulation, and other factors contribute to Long-covid^20,21^.

The primary aim of this systematic review and meta-analysis study was to evaluate the potential benefits of repurposing fluvoxamine to prevent clinical deterioration and death in COVID-19 patients, using data from global studies. Additionally, the study sought to identify key treatment parameters when initiating fluvoxamine treatment and explore its potential for mitigating Long-covid's long-term effects, including neurological, psychological, and physical symptoms.

## Methods

### Literature searches and selection

**Figure 1:**
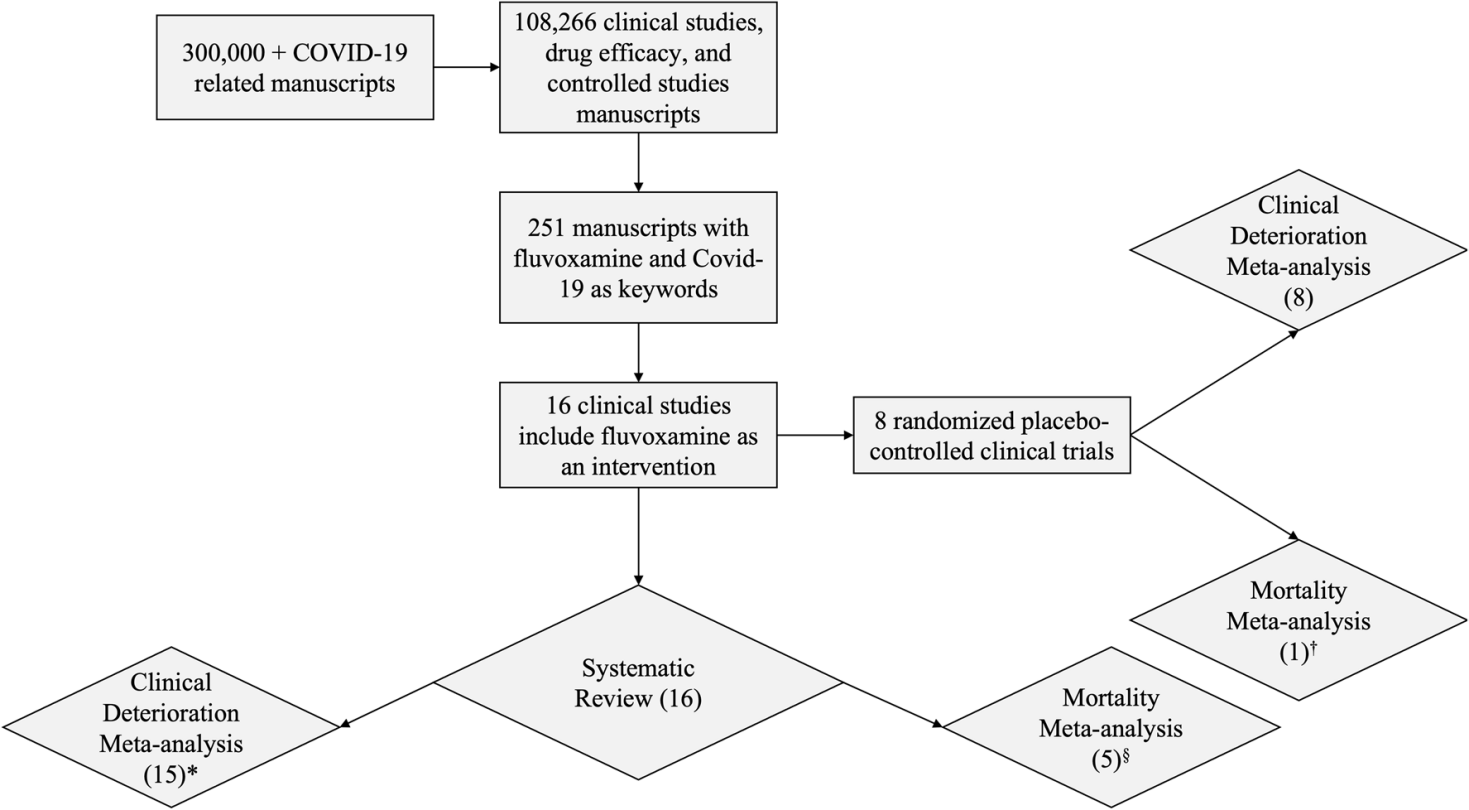
Study Selection. Studies were selected from various literature databases, identifying clinical trials with fluvoxamine for COVID-19. Studies were split into all studies and randomized placebo-controlled studies. *Oskotsky et al., retrospective trial included all SSRI drugs and combined fluvoxamine with fluoxetine and, therefore, was excluded from the meta-analysis. § Studies with no deaths in either group (fluvoxamine or control) were not entered into the COVID-19 mortality meta-analysis. †Only one placebo-controlled study of fluvoxamine in COVID-19 had deaths in either group (fluvoxamine or control), so the meta-analysis could not be carried out.

Since the outbreak of the COVID-19 pandemic in late 2019, there have been over 300,000 manuscripts (indexed in Scopus) that include keywords related to SARS-CoV-2, COVID-19, and coronavirus infection. Of these, 108,266 included keywords related to clinical studies, drug efficacy, and controlled studies. There are 251 manuscripts with fluvoxamine and COVID-19 as keywords. We screened the literature from databases including PubMed, Google Scholar, Scopus, and clinicaltrials.com using search terms related to fluvoxamine and COVID-19. We narrowed our search to include only the clinical trials, excluding preclinical studies and reviews (Fig. 1).

From our literature screening, 15 clinical studies include fluvoxamine as an intervention (Table 1). Of these, seven were randomized placebo-controlled clinical trials^12,22–27^. Seven were open-label "real world" studies^28–34^, and one was retrospective (Table 1)^35^. The Oskotsky et al., retrospective trial included all SSRI drugs^35^. Furthermore, there were very few fluvoxamine patients in the study, and fluvoxamine data was pooled with fluoxetine, so it was not included in our meta-analysis (Fig. 1).

**Table 1:** Summary of Clinical Studies on Fluvoxamine Included in the Systematic Review and Meta-Analysis. This table provides detailed information on all the clinical studies using fluvoxamine that were included in the systematic review and meta-analysis.

	Population	Study type	Dates	Intervention	Participants	Controls	Clinical deterioration	Death
STOP COVID Lenze et al	Adults with COVID 19 confirmed by PCR	Randomized placebo controlled	April 10, 2020, to August 5, 2020	100 mg of fluvoxamine or placebo 3 times daily for 15 days	80	72	Fluvoxamine 0/80 (0%) Control 6/72 (8.3%)	Fluvoxamine 0/80 (0%) Control 0 /72 (0%)
STOP COVID 2 Reiersen et al	Unvaccinated adults (30 + years) with COVID 19 confirmed by PCR	Randomized placebo controlled	December 22, 2020, to May 21, 2021	100 mg fluvoxamine or placebo twice daily for 15 days	272	275	Fluvoxamine 13/272 (4.8%) Control 15/275 (5.5%)	Fluvoxamine 0/272 (0%) Control 0/275 (%)
TOGETHER Reis et al	Adults with COVID 19 confirmed by PCR	Randomized placebo controlled	January 20, to August 5, 2021	100 mg fluvoxamine or placebo twice daily	741	756	Fluvoxamine 79/741 (11%) Control 119/756 (16%)	Fluvoxamine 1/548(0.2%) Control 12/618 (2%)
Covid OUT Bramante et al	Adults (30–85 years) with COVID 19 confirmed by PCR	Randomized placebo controlled	December 30, 2020, to February 14, 2022	50 mg fluvoxamine or placebo twice daily for 14 days	156	291	Fluvoxamine 15/156 (9.6%) Control 48/291 (16.5%)	Fluvoxamine 0/156 (0%) Control 0/291 (0%)
ACTIV-6 Arm B McCarthy et al	Adults with COVID 19 confirmed by PCR experiencing 2 or more symptoms of acute COVID-19 for 7 days or less	Randomized placebo controlled	August 6, 2021, to May 27, 2022,	50 mg fluvoxamine or placebo twice daily for 10 days	646	588	Fluvoxamine 27/646 (4.2%) Control 26/588 (4.4%)	Fluvoxamine 0/646 (0%) Control 0/588 (0%)
ACTIV-6 ARM E Stewart et al	Adults (30 + years) with COVID 19 confirmed by PCR experiencing 2 or more symptoms of acute COVID-19 for 7 days or less	Randomized placebo controlled	August 25, 2022, and January 20, 2023	100 mg of fluvoxamine or placebo twice daily for 13 days	589	586	Fluvoxamine 14/589 (2.3%) Control 21/586 (3.6%)	Fluvoxamine 0/589 (0%) Control 0/586 (0%)
Soe et al	Adults (18 + years) with COVID 19 confirmed by PCR	Randomized placebo controlled	January 15, 2021, to February 19, 2021	100 mg of fluvoxamine or placebo twice daily for 10 days	26	26	Fluvoxamine 2/26 (7.6%) Control 2/26 (7.6%)	N/A
Calusic et al	Adults (18 + years) with COVID 19 confirmed by PCR	Open-label, prospective cohort trial with matched controls	April 1, to May 31, 2021	100 mg three times daily	51	51	Fluvoxamine 30/51 (58.8%) Control 39/51 (76.5%)	Fluvoxamine 30/51(58.8%) Control 39/51(76.5%)
Pineda et al	Patients (15 + years) COVID 19 confirmed by PCR or antigen test kit	Prospective observational real-world study	November 1, 2020 toJanuary 31, 2022	100 mg two or three times a day (tolerance depending)	594	63	Fluvoxamine 30/594 (5%) Control 10/63 (16%)	Fluvoxamine 1/594 (0.2%) Control 4/63 (6.3%)
Seftel et al	Adults with COVID 19 confirmed by PCR	Prospective cohort observational real-word study	November 1, to December 31, 2020	50 mg twice daily for 14 days	65	48	Fluvoxamine 0/65 (0%) Control 6/48 (12.5%)	Fluvoxamine 0/65 (0%) Control 1/48 (2.1%)
Kirenga et al	Adults with COVID 19 confirmed by PCR	prospective interventional open-label cohort study	December 1, 2021 to February 28, 2022	100 mg twice a day for 10 days	94	222	Fluvoxamine 43/94 (45.7%) Control 153/222 (68.9%)	Fluvoxamine 29/94 (30.9%) Control 126 /222 (56.8%)
Wannigama et al	Adults (18–60 years) with COVID 19 confirmed by PCR	open-label, multi-arm, randomized controlled trial	October 1, 2021, to September 21, 2022	100–150 mg over 14 days	132	366	Fluvoxamine + standard care 9/163 (5.5%) Control 42/366 (11.5%)	Fluvoxamine + standard care 0/162 (0%) Control 0/336 (0%)
Siripongboonsitti et al	Adults (18 + years) with COVID 19 confirmed by PCR	Open-label randomized controlled trial	June 26, 2021, to February 22, 2022	50 mg Fluvoxamine 2 times per day + favipiravir	132	134	Fluvoxamine 4/132 (3%) Control 0/134 (0%)	N/A
Tsiakalos et al	Adults with COVID 19 confirmed by PCR	Real-World, Retrospective, before–after Analysis	September 2021 and December 2021	100 mg fluvoxamine 2 tmes daily	53	50	Fluvoxamine 2/53 (3.8%) Control 8/50 (16%)	Fluvoxamine 0/53 (0%) Control 0/50 (0%)
Oskotsky et al	Adults COVID 19 confirmed by PCR or laboratory antigen test kit	Retrospective cohort study	January to September 2020	N/A	481	7215	N/A	Fluvoxamine/fluoxetine 48/481 (10.0%) Control 956/7215 (13.3%)

### Literature quality scoring

The clinical studies identified by this manuscript were scored based on a set of questions previously devised for the literature quality scoring^36^, expanded from the JADAD scoring system for clinical trials^37^. Each question carried 1 point for a positive answer, and 1 point was subtracted for a negative answer. The maximum score was 12, and the minimum was -11 (question 2 depended on a positive answer to question 1). The quality scoring questions were as follows:

1. Was the study randomized?
2. If yes to question 1, was the randomization appropriate?
3. Was the study double-blinded?
4. Was there a description of any withdrawals from the study?
5. Was there a clear description of the inclusion/exclusion criteria for the study?
6. Was there an appropriate control group?
7. Was the dose appropriate?
8. Were the adverse effects monitored and described?
9. Was the method of statistical analysis described?
10. Was there an appropriate follow-up of patients?
11. Are the primary and secondary outcomes clearly defined?
12. Have the results of the study been published?

A positive outcome in the scoring was considered appropriate for entering data into the meta-analysis. For the quality scoring, the Active-6 studies were assessed as one study.

### Data collection and statistical analysis

All eligible manuscripts and data sets available before the end of January 2024 were selected and included in the study.

Data were extracted from the eligible publications or data sets, and the meta-analysis was carried out using Jamovi computer software (Version 2.4.8.0 for Mac) (https://www.jamovi.org)^38^, and IBM SPSS Statistics (Version 29.0.1.0 (171) for Mac) (https://www.ibm.com/products/spss-statistics). In the analysis of mortality, we only included studies that had a death in at least one of the groups (fluvoxamine or standard care/placebo) to avoid bias caused by the different group sizes. The estimated mean difference, 95% CI, and *p* value were presented. Heterogeneity statistics were presented with the I^2^ value and its corresponding *p* value. Data was presented as a Forrest plot showing each study's weighted log odds ratio and the estimated mean difference for the combined studies. Publication bias was assessed using the Rosenthal approach to the Fail-safe N (file draw analysis) and presented with a funnel plot. The analysis used the log odds ratio as the outcome measure, and a random-effects model was fitted to the data. The amount of heterogeneity (i.e., tau^2^) was estimated using the restricted maximum-likelihood estimator^39^. In addition to the estimate of tau^2^, the Q-test for heterogeneity and the I^2^ statistics were calculated. If any amount of heterogeneity was detected (i.e., tau^2^ > 0, regardless of the results of the Q-test), a prediction interval for the true outcomes was also provided. Studentized residuals and Cook's distances were used to examine whether studies may be outliers and/or influential in the model context. Studies with a Cook's distance larger than the median plus six times the interquartile range of the Cook's distances were considered influential. The rank correlation test and the regression test, using the standard error of the observed outcomes as predictors, were used to check for funnel plot asymmetry.

## Results

### Qualitative analysis

The quality of the studies identified within the manuscript was assessed with the 12 quality scoring questions (Supplementary Table 1). All the studies identified for this meta-study scored one or higher and were therefore deemed eligible for meta-analysis. Four of the studies scored below five^28,29,32,35^.

#### Placebo-controlled trials

Of the 16 studies identified by this analysis, eight were placebo-controlled studies with acceptable blinding methods. The STOP COVID trial (NCT04342663)^12^ was one of the initial trials to investigate the repurposing of fluvoxamine to prevent clinical deterioration of patients infected with COVID-19. It was relatively small, with 80 patients in the fluvoxamine group (300 mg/day) and 72 in the placebo group. The study investigated only the clinical worsening of the disease over 15 days. Of the 80 patients in the fluvoxamine group, there was no clinical worsening.

In contrast, only six patients of the 72 taking placebo had clinical worsening: two had shortness of breath with oxygen saturation < 92% but no need for oxygen, three had oxygen saturation < 92% and required supplemental oxygen and were hospitalized related to dyspnea or hypoxia, and one patient had an oxygen saturation of < 92%, required supplemental oxygen, was hospitalized related to dyspnea or ​hypoxia and was on a ventilator for three days. None of the patients in either group died. The authors of this study acknowledged the limitations of the study as being small and the outcomes possibly being influenced by the initial oxygen saturation of the patients. During the STOP COVID trial, the investigators added questionnaires about Long-covid symptoms, which were to be completed several months after the trial ended. Still, the details had not been published in a peer-reviewed manuscript at the time of this review.

Another trial early in the pandemic was the Together trial (NCT04727424)^22^. This trial was considerably larger than the STOP COVID trial, with 741 patients allocated to fluvoxamine (200 mg/day) and 756 to placebo. The study showed that 11% of patients in the fluvoxamine group and 16% of patients in the placebo group required observation for COVID-19 in an emergency setting for more than six hours or were transferred to a tertiary hospital. Furthermore, there were 17 deaths in the fluvoxamine group and 25 deaths in the placebo group.

The STOP COVID 2 (NCT04668950) trial was initiated in late 2020 and followed up on the initial apparent success of the smaller STOP COVID trial. This study investigated a lower dose of fluvoxamine (100 mg two times daily) than the first STOP COVID trial in 272 patients, compared to 275 patients taking the placebo. The number of patients with clinical deterioration within 15 days in the fluvoxamine group was 13, and in the placebo group, it was 15. This study planned a secondary outcome for post-COVID follow-up for 90 days. However, the data for such a study had not been posted or published at the time of this review. The STOP COVID 2 investigators planned to combine this follow-up data with the initial STOP COVID data to give more power for analysis, and these results were published after the completion of this review^40^.

A further trial started in late 2020, the COVID OUT trial (NCT04510194). This study used fluvoxamine alone and in combination with metformin, ivermectin, or metformin vs placebo. The study reports the composite data of fluvoxamine (including coadministration with the other drugs)^23^.

The study reported that 79 of 329 patients had clinical deterioration after treatment with fluvoxamine (with or without another drug), and 80 out of 321 had clinical deterioration after the placebo. This resulted in an odds ratio of 0.96 (95% CI 0.6811 to 1.3629) and a z-statistic of 0.2 and *p* = 0.833. However, the other drugs in the study could be masking fluvoxamine's effects. Furthermore, as the study mentions in its limitations, the dose was relatively low compared to the other studies. We extracted the clinical deterioration data for the patients who only received fluvoxamine (15/156, 9.61%) and placebo (48/291, 21.92%), resulting in an odds ratio of 0.58 (95% CI 0.3163 to 1.0745), a z-statistic of 1.73 and *p* = 0.08 (suggesting clinical detonation was potentially more likely in the placebo group compared to the fluvoxamine group).

The study by Soe et al*.*^25^, commenced in January 2021, treating COVID-19 patients with fluvoxamine or placebo. The study reported that two patients in each group (placebo (2/26) and fluvoxamine (2/26)) had clinical deterioration and concluded that fluvoxamine had no significant effect on the prevention of clinical deterioration. However, the study was terminated early as the community treatment center was closed as the number of patients with COVID-19 infections reduced. The number of patients enrolled did not meet the intended minimum number of subjects, so *post-hoc* power was very low to give statistical significance.

The ACTIV-6 platform (NCT04885530) investigated multiple drugs for the potential repurposing against COVID-19. The study covered two doses of fluvoxamine: The low dose, 50 mg twice per day (ARM B- NCT05890586), and the high dose, 100 mg twice daily (ARM E- NCT05894564).

The low-dose ACTIV-6 study of fluvoxamine (ACTIV-6 ARM B- NCT05890586)^24^ took place between August 6, 2021, and May 27, 2022, 1331 participants were randomized, and 1288 completed the trial (646 in the fluvoxamine group (50 mg twice per day) and 588 in the placebo group). The primary outcome of this study was time to sustained recovery. However, this is a problematic outcome measure, as it relies on an accurate estimation of when symptoms start and stop, which is subjective. The trial also included secondary outcomes such as hospitalization, clinical deterioration, and death. The study resulted in 12 days to sustained recovery for the fluvoxamine group and 13 days for the placebo. This difference was not statistically significant.

Furthermore, in the secondary outcomes of clinical deterioration (healthcare utilization events defined as hospitalizations and emergency care visits), fluvoxamine resulted in 27 out of 646, whereas placebo resulted in 26 out of 588, and there were no deaths in either group. This study did not find any significant benefits for fluvoxamine. However, as it was a broadly inclusive study population, few clinical events were observed; thus, they could not study the effects of treatment on clinical outcomes such as hospitalization. Furthermore, the time to treatment was particularly slow, with treatment occurring within ten days (the median time being five days). Other studies we have covered in this analysis initiated therapy within two to seven days.

The high dose (100 mg twice daily) ACTIV-6 study (ACTIV-6 ARM E- NCT05894564)^26^ occurred between August 25, 2022, and January 20, 2023. A total of 589 participants were randomized to receive fluvoxamine, and 586 participants were randomized to placebo. Similarly, to the low-dose study^24^ the primary outcome was time to sustained recovery, which, as we have mentioned, is problematic as a study outcome. As with the low dose^24^, differences in time to sustained recovery were not observed.

The higher dose study also used the time to recovery metric as an endpoint. However, the study also reports the healthcare events, plus identified that 14 out of 589 in the fluvoxamine group, compared with 21 out of 586 in the placebo group, experienced healthcare utilization events defined as hospitalization, emergency department/urgent care visits. Again, no deaths were reported in either group.

#### Open-label studies

Of the 15 studies identified by this analysis, six were open-label/real-world studies, where fluoxetine-treated patients were compared to matched patients who declined to take fluvoxamine. One of the earlier studies of fluvoxamine was an open-label cohort study^30^. In the Seftel et al*.* study hospitalization was 0% (0 of 65) with fluvoxamine and 12.5% (6 of 48) with observation alone. Calusic et al.^28^, investigated the effects of fluvoxamine in COVID-19 ICU patients and did not find that fluvoxamine reduced time in ICU or time on ventilators. However, they did find a statistically significant improvement in mortality in the fluvoxamine-treated group (58.8% (n = 30/51)) compared to the control group (76.5% (n = 39/51)). A larger study carried out in Honduras^29^, showed similar results in preventing clinical deterioration (fluvoxamine, 30/594 (5%); standard care 10/63 (16%)) and death (fluvoxamine 1/594 (0.2%); standard care 4/63 (6.3%)). A Ugandan study of fluvoxamine showed that it was significantly associated with reduced mortality but did not decrease time spent in hospital^31^. A small open-label study in Greece investigated fluvoxamine as part of a real-world, retrospective, before–after analysis, and the results also indicated that it reduced the risk of clinical deterioration in COVID-patients (3.8% of fluvoxamine-treated patients and 16% standard care patients experienced clinical deterioration (OR 0.12; 95% CI 0.02–0.70, *p* < 0.02)^32^.

A 2021 study in Thailand investigating multiple drugs, alone and in combination, revealed that 0/162 (0%) patients taking fluvoxamine alone experienced clinical deterioration by day 9, whereas 227/336 (67.5%) receiving standard care experienced clinical deterioration requiring hospitalization, and in total through the whole 28 days of study 9/162 experienced some form of clinical decline in the fluvoxamine group and 321/336 in the standard care group (NCT05087381)^33^.

Finally, another study in Thailand, between June 2021 and February 2022 (Thai Clinical Trials Registry (TCTR) no. 20210615002), compared favipiravir to fluvoxamine plus favipiravir in mild COVID patients. The study showed that after five days of treatment, there was 0/134 (0%) clinical deterioration in the favipiravir group, whereas 4/132 patients in the fluvoxamine plus favipiravir group with no statistical difference between the two groups^34^.

#### Retrospective studies

Of the 15 studies trialing fluvoxamine as an intervention for COVID-19 infection, one was a retrospective study matching SSRI-taking patients to age-matched controls showed that fluoxetine or fluvoxamine resulted in a lower incidence of death of COVID compared to the controls (48 of 481 (10.0%) vs 956 of 7215 (13.3%) with a relative risk of 0.74 (95% CI 0.55–0.99), (*p* = 0.04). However, this study did not separate fluvoxamine from fluoxetine patients in the analysis, although the study did find that any SSRI was linked to a reduced risk of death from COVID-19^35^.

#### Long-covid studies

One of the studies above has published results of Long-covid diagnoses in patients treated with fluvoxamine as part of the COVID OUT study (NCT04510194). At a ten-month follow-up after randomization, 7.5% (95% CI 4.4% to 10.5%) of patients reported a Long-covid diagnosis in the blinded control group, whereas 10.1% (95% CI 6.6% to 13.5%) of the fluvoxamine group. Fluvoxamine did not decrease the likelihood of being diagnosed with Long-covid, which is consistent with outcomes in the first 14 days of the COVID OUT trial^41^.

Another placebo-controlled double-blinded study investigating the neuropsychiatric symptoms of post-COVID syndrome in mild to moderate patients^42^. A total of 42 patients who received fluvoxamine, and 53 patients who received placebo completed the study. Neuropsychological symptoms of post-COVID-19 syndrome were present in 76.2% and 81.4% of patients in fluvoxamine and placebo groups, respectively. 9/42 (21.4%) fluvoxamine-treated patients suffered from fatigue, whereas in the placebo group, it was 19/43 (44.2%) (*p* = 0.026, RR = 0.48 (95% CI 0.25–0.95). Unsurprisingly, given the pharmacological nature of fluvoxamine, there was also a reduction in reported depression in the fluvoxamine group compared to the placebo 1/42 (2.4%) and 7/43 (16.3%) (*p* = 0.058 RR0.114 (95% CI 0.02–1.13).

In the STOP COVID 1 & 2 trials^12,27^ collected follow-up data regarding the degree of recovery toward baseline and persistent COVID-19-related symptoms several months after the acute COVID-19 clinical trial periods. These results were not yet published at the time of the current review. Still, some information was obtained from the STOP COVID investigators (including AMR, co-author of the current manuscript), and part of these results have now been published^40^. Regardless of treatment condition, most trial participants in each STOP COVID trial reported being less than 100% recovered back to usual health at follow-up. Fluvoxamine did not appear to affect this, but those who took fluvoxamine during the acute COVID-19 trial were about half as likely to report being less than 60% recovered (statistically significant only when follow-up data from both trials are combined). Individual symptom-level Long-covid data from both trials is still undergoing analysis^40^.

A retrospective analysis of patients taking SSRIs at the time of infection showed a 29% reduction (*p* = 0.0004) in the relative risk of Long-covid among patients (n = 1521) receiving an SSRI with σ1R agonism (fluvoxamine, fluoxetine, escitalopram) and a 21%  reduction (p = 0.005) in the relative risk of Long-covid among patients (n = 1803) taking an SSRI without σ1R agonism (citalopram, paroxetine, sertraline) compared with patients (n = 14,584) not taking an SSRI^43^. As pointed out by K. Hashimoto^44^ a preprint of this study included citalopram as an SSRI with σ1R activity, which other studies have suggested as incorrect as it does not potentiate NGF-induced neurite outgrowth in PC-12 cells^45^. Other studies have suggested that SSRIs could have the potential for treatment of Long-covid^46,47^ due to the anti-inflammatory activity in the treatment of depression in patients with Long-covid^48^.

## Meta-analysis

### Fluvoxamine to prevent clinical deterioration in COVID-19 infection (all 14 studies)

Initially, we examined every study where fluvoxamine was used to treat COVID-19 and clinical deterioration was reported. Clinical deterioration was defined as the requirement for hospital admission for any reason after a positive test for COVID-19 (for the studies taking place within a hospital setting, it was the requirement of treatment other than observation).

**Figure 2:**
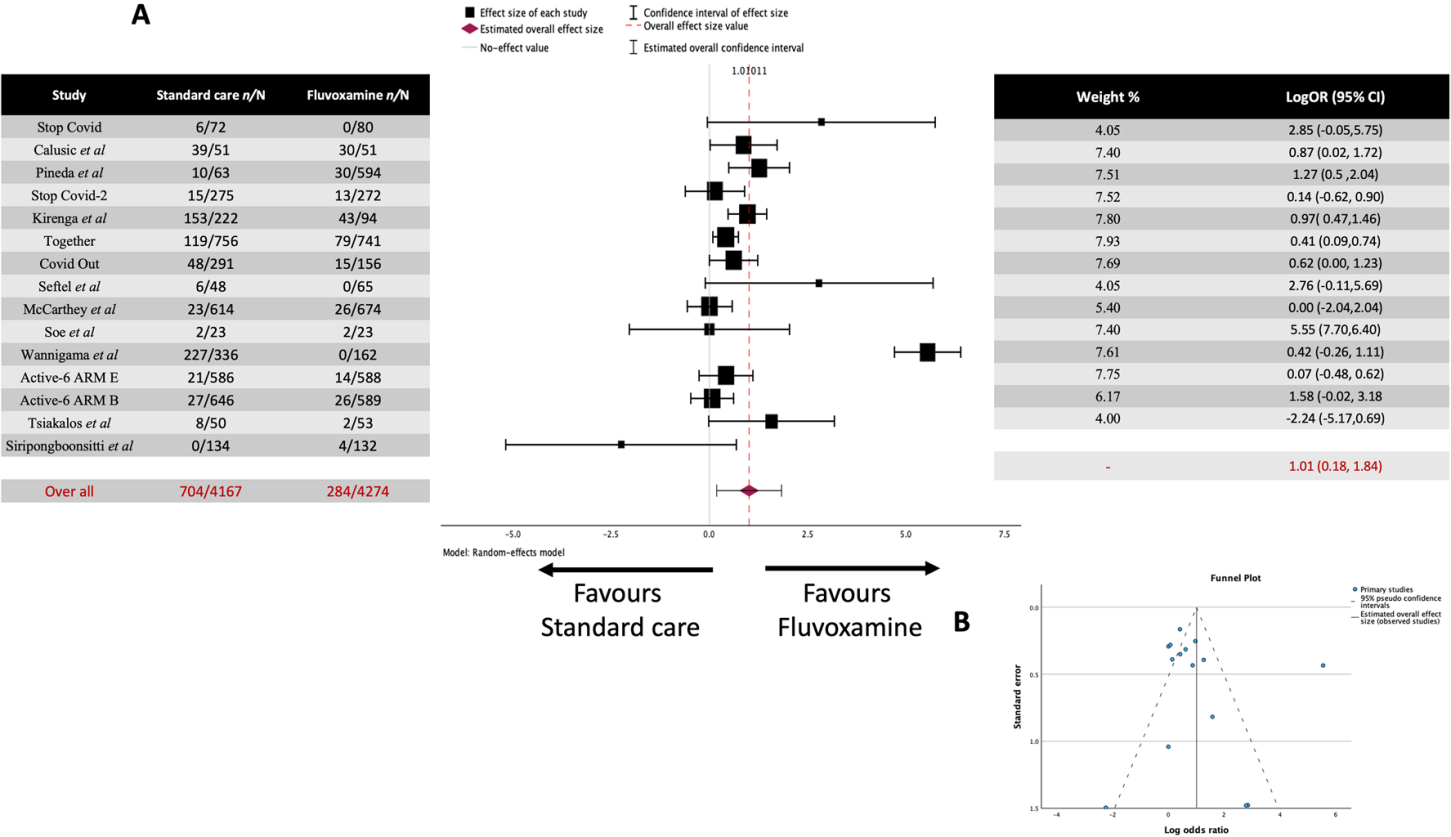
Meta-analysis for every study regarding fluvoxamine treatment of COVID-19 (**A**) Forrest plot. The Log OR was 1.087 (95% CI 0.200 to 1.973) z = 2.402, *p* = 0.016. (**B**) Funnel Plot. Rank correlation (*p* = 0.1925) and regression test (*p* = 0.9398) do not indicate any funnel plot asymmetry.

A total of 14 studies were included in the analysis (Fig. 2A), covering 7153 patients. Among this, 681 out of 3553 (19.17%) in the standard care group and 255 out of 3600 (7.08%) in the fluvoxamine-treated group experienced clinical deterioration.

The observed log odds ratios ranged from -2.2429 to 5.8966, with most (79%) estimates being positive. The estimated average log odds ratio based on the random-effects model was 1.087 (95% CI 0.200 to 1.973). Therefore, the average outcome differed significantly from zero, indicating a benefit from fluvoxamine over placebo/standard care (z = 2.402, *p* = 0.016).

The Q-test for heterogeneity was not significant, but some heterogeneity may still be present in the true outcomes (Q(13) = 170.4235, *p* < 0.0001, tau^2^ = 2.7003, I^2^ = 94.9916%). A 95% prediction interval for the true outcomes is given by -1.6431 to 3.7587. Hence, although the average outcome is estimated to be positive, in some studies, the true outcome may be negative. An examination of the studentized residuals revealed that one study (NCT05087381)^33^ had a value larger than ± 2.9137 and maybe a potential outlier in the context of this model. According to the Cook's distances, one study (NCT05087381)^33^ could be overly influential. Neither the rank correlation nor the regression test indicated any funnel plot asymmetry (Fig. 2B) (*p* = 0.1925 and *p* = 0.9398, respectively).

### Fluvoxamine to prevent clinical deterioration in COVID-19 infection (placebo-controlled)

Open-label studies, as the name suggests, do not contain a masking protocol, and there is no placebo control, as there is an inherent risk of the placebo effect in these studies, even when matched to the standard care controls who chose not to take fluvoxamine. Therefore, we also conducted the meta-analysis using only the gold standard placebo-controlled double-blind studies.

**Figure 3:**
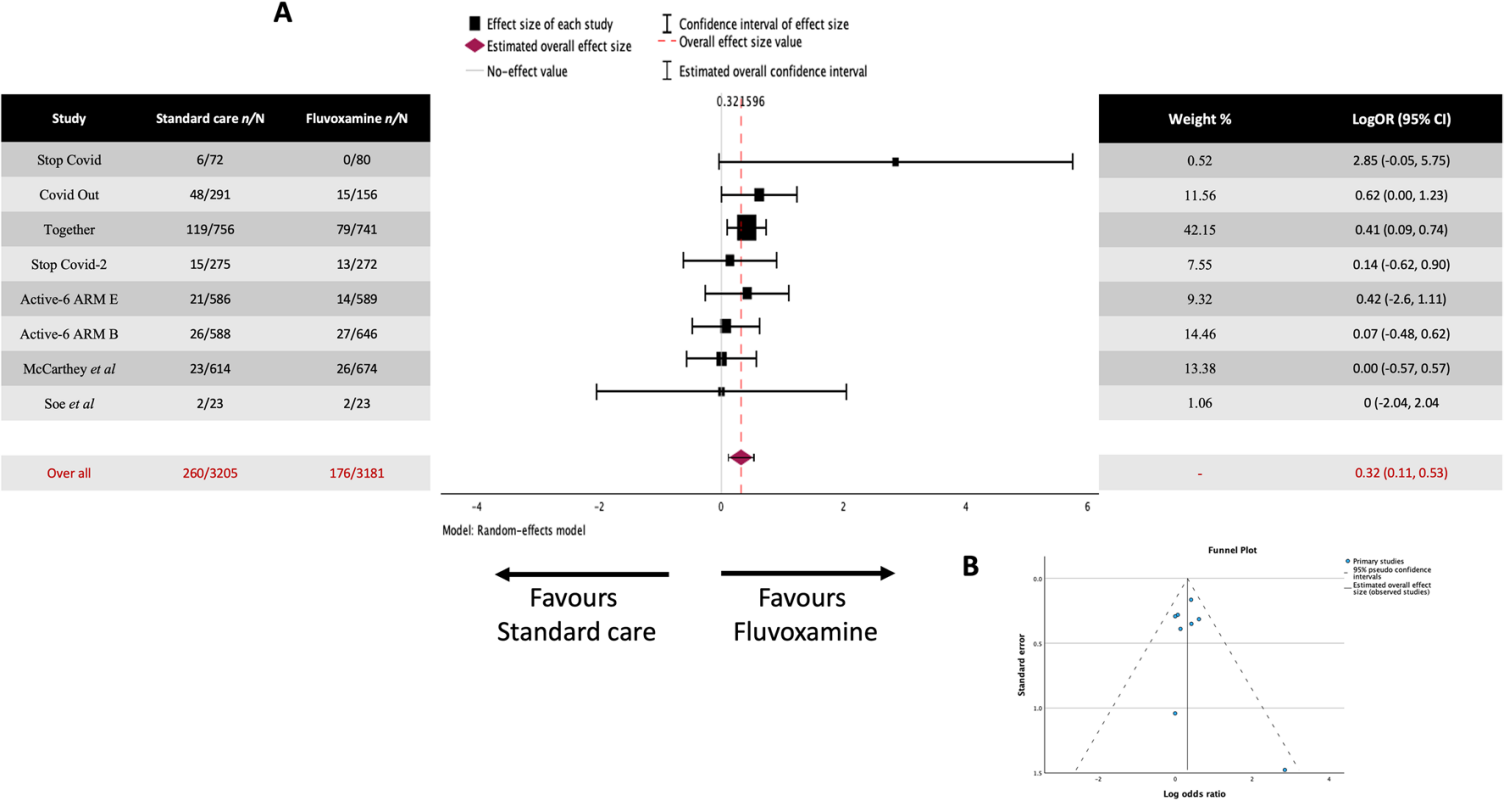
Meta-analysis for the placebo-controlled studies on fluvoxamine treatment of COVID-19 and clinical deterioration. (**A**) Forrest plot. LogOR 0.359 (95% CI 0.1111 to 0.5294), which differs significantly from zero (z = 3.103, *p* = 0.002) (**B**) Funnel Plot. Rank correlation (*p* = 0.5619) and the regression test *p* = 0.4018 do not indicate any asymmetry.

Seven studies were included in the analysis (Fig. 3A) covering 5080 patients. 237 out of 2573 (9.21%) in the standard care and 150 out of 2507 (5.98%) in the fluvoxamine-treated group suffered clinical deterioration.

The observed log odds ratios ranged from -0.2171 to 2.8506, with most estimates being positive (71%). The estimated average log odds ratio based on the random-effects model was 0.359 (95% CI 0.1111 to 0.5294). Therefore, the average outcome differed significantly from zero, indicating a benefit from fluvoxamine over placebo/standard care (z = 3.103, *p* = 0.002). The Q-test showed no significant heterogeneity in the true outcomes (Q(6) = 8.3037, *p* = 0.2167, tau^2^ = 0.0198, I^2^ = 14.6071%). A 95% prediction interval for the true outcomes is given by -0.0360 to 0.7330. Hence, even though there may be some heterogeneity, the true outcomes of the studies are generally in the same direction as the estimated average outcome.

The studentized residuals showed that none of the studies had a value larger than ± 2.6901, so there is no indication of outliers in the context of this model. According to the Cook's distances, none of the studies are overly influential. Neither the rank correlation nor the regression test indicated any asymmetry in the funnel plot (Fig. 3B) (*p* = 0.5619 and *p* = 0.4018, respectively).

### Fluvoxamine in every study that included COVID-19 caused mortality

COVID-related mortality was also an important metric that should be studied in treating COVID-19 with fluvoxamine, particularly in the early stages of the outbreak. We initially performed a meta-analysis of all the studies that reported COVID-related deaths in the study. A total of 12 studies measured death as an outcome of COVID-19 infection, covering 7722 patients. A total of 182 out of 3791 (4.8%) in the standard care group and 61 out of 3931 (1.56%) in the fluvoxamine group died because of COVID-19 infection.

**Figure 4:**
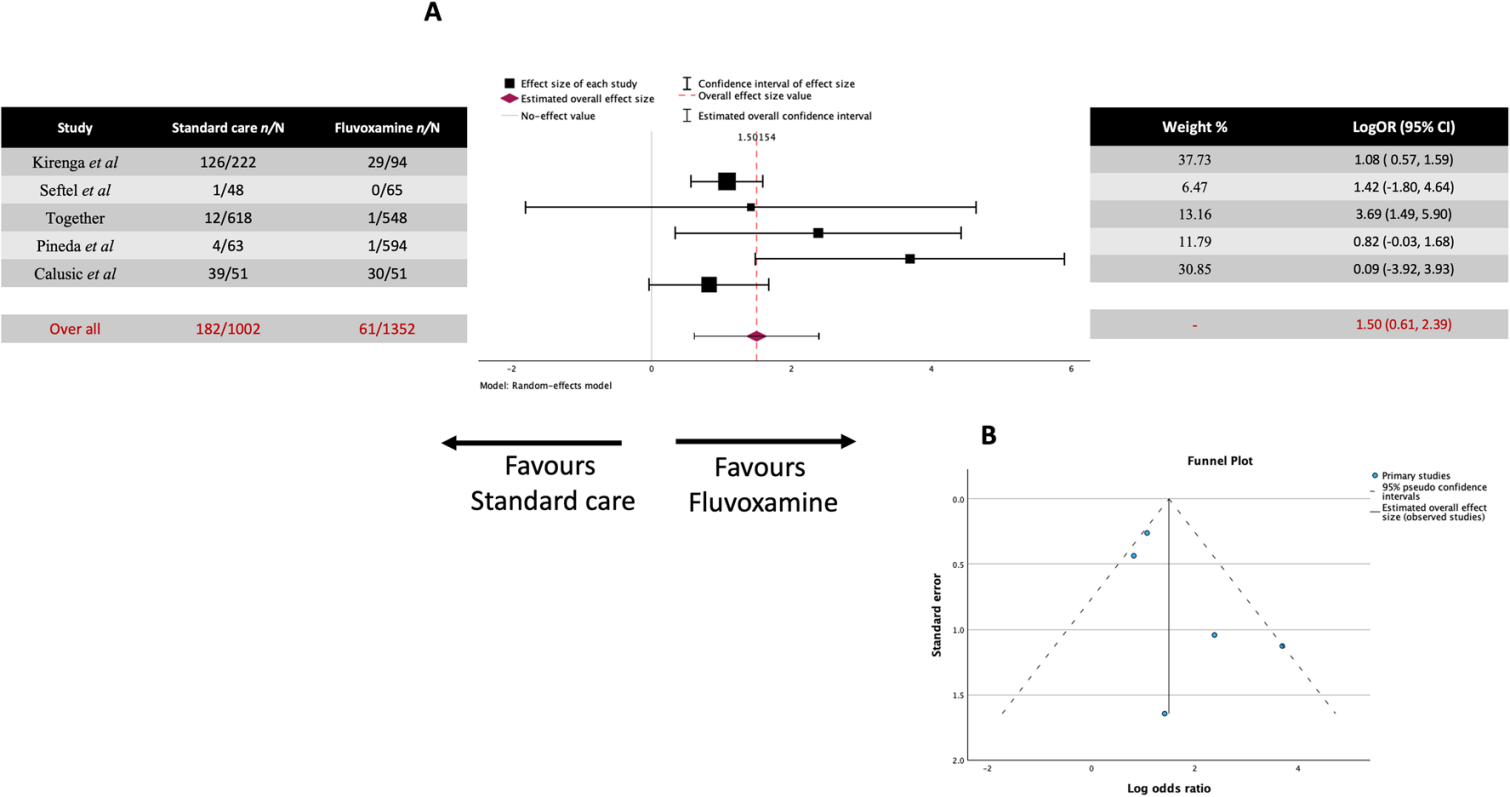
Meta-analysis for all the studies carried out regarding fluvoxamine treatment and COVID-19 mortality. (**A**) Forrest plot. LogOR 1.502 (95% CI 0.621 to 2.391) z = 3.301, *p* < 0.001. (**B**) Funnel Plot. The rank correlation *p* = 0.0863 and the regression test indicated *p* = 0.0629 did not reveal any plot asymmetry.

However, only five of the studies recorded a death in one of the groups (fluvoxamine or standard care); thus, only these studies were included in the meta-analysis (Fig. 4A). The observed log odds ratios ranged from 0.8220 to 3.6940, with all the estimates being positive. The estimated average log odds ratio based on the random-effects model was 1.502 (95% CI 0.621 to 2.391). Therefore, the average outcome differed significantly from zero, suggesting fluvoxamine to be beneficial over placebo/standard care (z = 3.301, *p* < 0.001). According to the Q-test, there was no significant heterogeneity in the true outcomes (Q(4) = 7.1562, *p* = 0.1279, tau^2^ = 0.4772, I^2^ = 56.5333%). A 95% prediction interval for the true outcomes was given by -0.1182 to 3.1213. Hence, although the average outcome was estimated to be positive, in some studies, the true outcome may be negative. One study Kirenga et al*.*^31^ had a relatively large weight compared to the rest (i.e., a weight at least three times as large as weights across the studies). An examination of the studentized residuals revealed that none of the studies had a value larger than ± 2.5758. Hence, there was no indication of outliers in the context of this model. According to the Cook's distances, none of the studies are overly influential. Neither the rank correlation nor the regression test indicated any asymmetry in the funnel plot (Fig. 4B) (*p* = 0.4833 and *p* = 0.0629, respectively).

### Fluvoxamine in every placebo-controlled study that included COVID-19 caused mortality

As mentioned above, we also performed the meta-analysis with clinical deterioration, including only those placebo-controlled studies. A total of seven placebo-controlled trials that studied death as an outcome of COVID-19 infection covered 6036 patients. A total of 12 patients out of 3071 (0.39%) in the standard care group and 1 out of 2965 (0.025%) in the fluvoxamine group died from COVID-19 infection. However, only one placebo-controlled study observed any deaths. Thus, a meta-analysis cannot be done.

## *Meta*-analysis-subgrouping analysis

At the start of this study, we set out to discover if certain aspects of fluvoxamine treatment for COVID-19, such as dose or time to intervention, affected the study's outcome. Furthermore, there were some indications of heterogeneity in the above meta-analysis. Therefore, we have carried out subgrouping analysis, splitting the data into groupings as follows: High (200 mg or more/day) and low dose (less than 200 mg) (we also further divided the doses into up to 300 mg/ day, up to 200 mg/day and up to 100 mg/day) and time to intervention, early (within three days) and medium (within four to seven days) and late (within eight to ten days). We chose the grouping based on the observation that the low-dose studies tended to be the ones that were less likely to result in a positive outcome for fluvoxamine. We chose time for treatment as, early in the pandemic, it became clear that the second week of infection was where clinical deterioration became apparent, and at that point, starting drug treatment became less effective.

### Fluvoxamine high/low dose subgroup analysis in clinical deterioration

**Figure 5:**
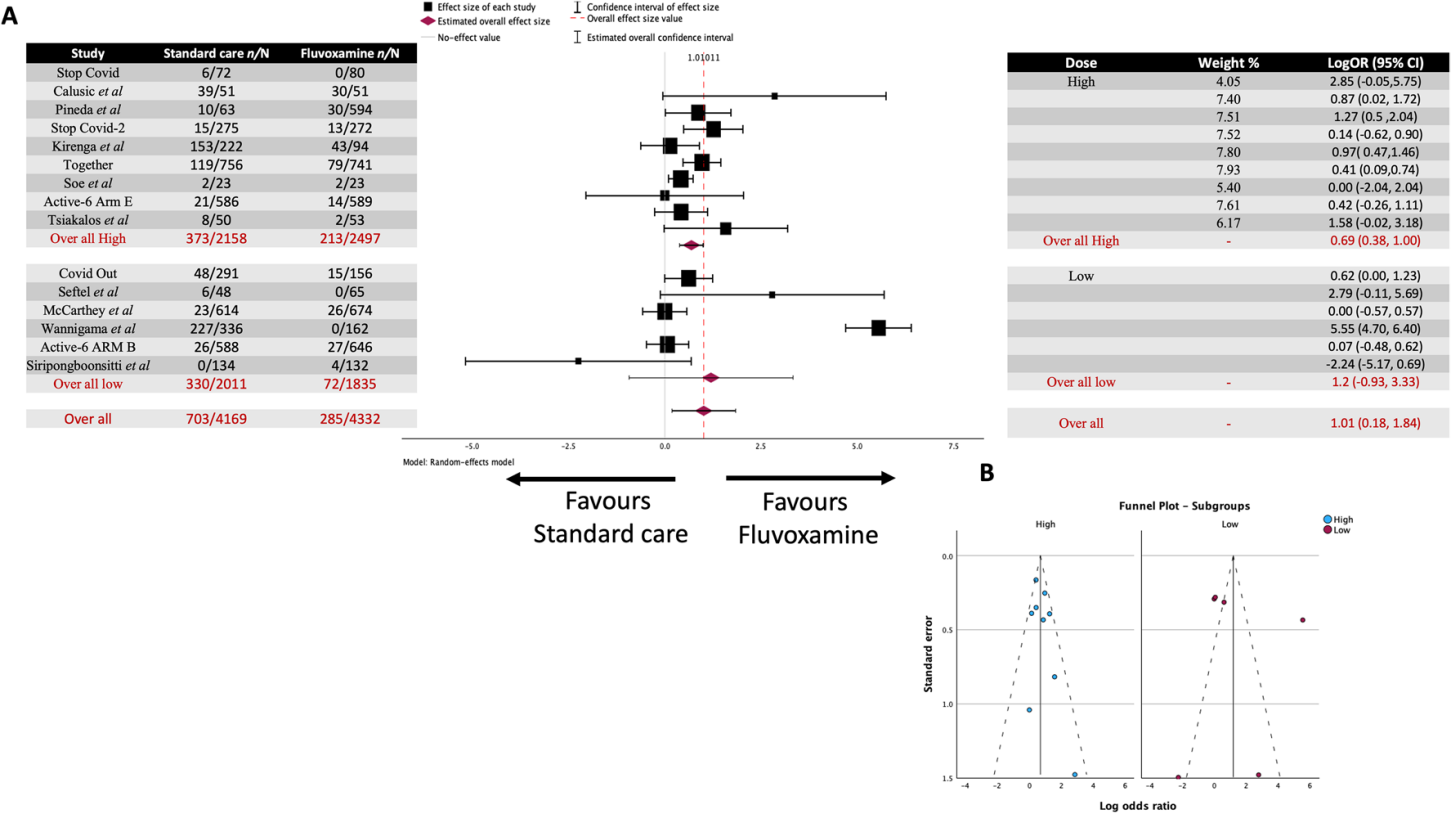
Meta-analysis for all the studies on fluvoxamine treatment of COVID-19 and clinical deterioration sub-grouped by dose. (**A**) Forrest plot. High dose LogOR 0.6957 (95% CI 0.3777 to 1.0137) z = 4.2882, *p* < 0.0001. Low dose LogOR 1.198 (95% CI -0.930 to 3.325) z = 1.082, *p* = 0.279. (**B**) Funnel Plot. No Asymmetry was observed at either high or low dose grouping.

**Figure 6:**
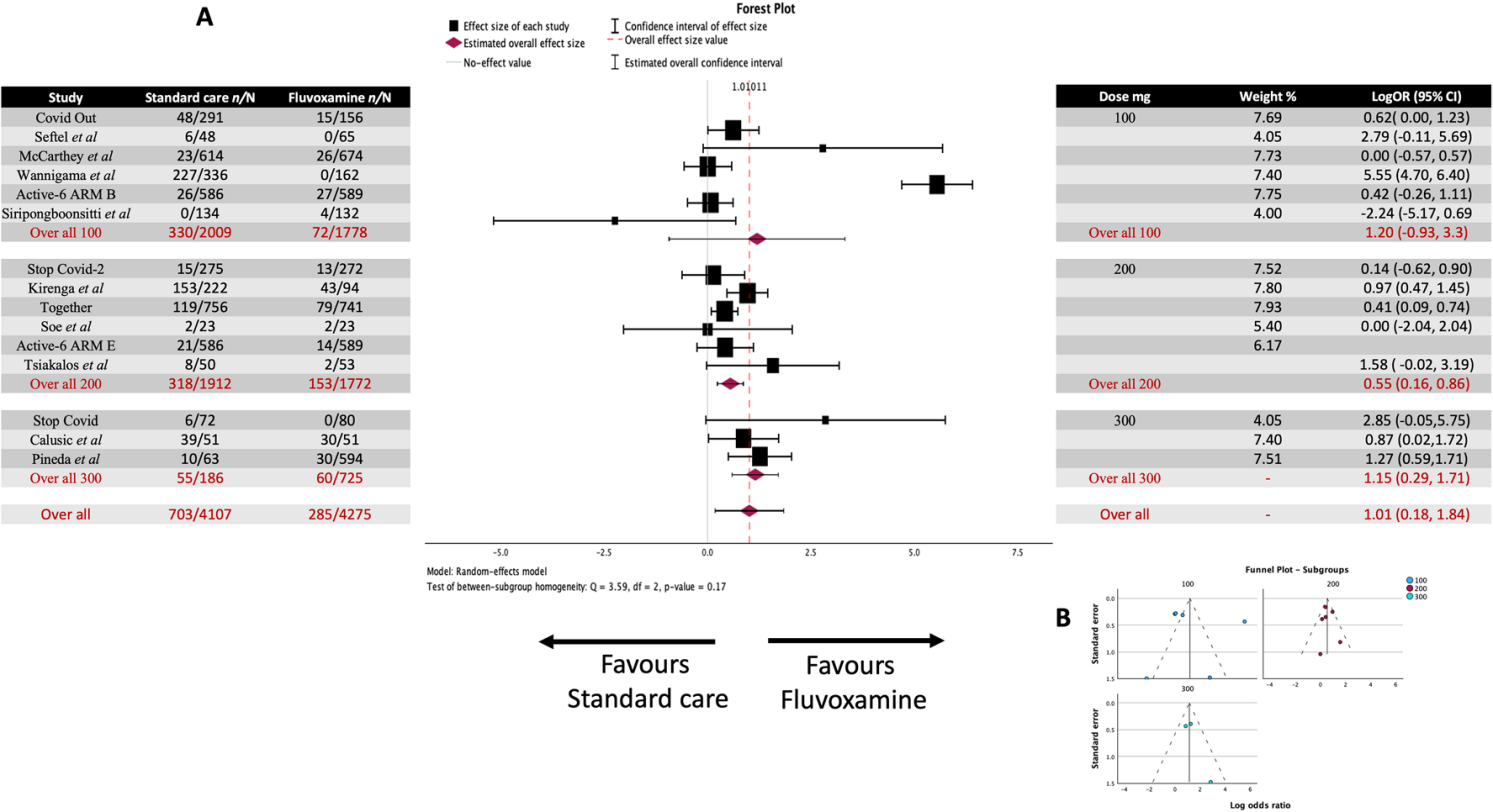
Meta-analysis of sub-grouped Fluvoxamine/COVID-19 studies based on dose, up to 100, up to 200, and up to 300 mg/day. (**A**) Forrest plot. 100 mg/day LogOR 1.43 (95% CI -1.16 to 4.01) z = 1.082, *p* = 0.279. 200 mg/day LogOR 0.550 (95% CI 0.236 to 0.865) z = 3.433 *p* < 0.001. 300 mg/day LogOR 1.153 (95% CI 0.593 to 1.713) z = 4.035 *p* < 0.001 (**B**) Funnel plots. No Asymmetry was observed at dose grouping.

Nine studies employed a high dose (≥ 200 mg/day), and five used a low dose (< 200 mg/day). These were grouped and analyzed (Figs. 5 and 6), covering 4655 patients. 373 from 2158 (17.2%) in the standard care group and 213 from 2497 (8.5%) in the fluvoxamine group succumbed to clinical deterioration.

In the nine high-dose studies, the observed log odds ratios ranged from 0.0000 to 2.8506, with most being positive estimates (89%). The estimated average log odds ratio based on the random-effects model was 0.6957 (95% CI 0.3777 to 1.0137). Therefore, the average outcome differed significantly from zero (z = 4.2882, *p* < 0.0001). Neither the rank correlation nor the regression test indicated any funnel plot (Fig. 6B) asymmetry (*p* = 0.2595 and *p* = 0.2038, respectively).

A total of five studies were included in the low-dose subgroup analysis, covering a total of 2655 patients. 307 from 1397 (21.97%) in the standard care group and 46 from 1246 (3.69%) in the fluvoxamine group suffered clinical deterioration. The observed log odds ratios ranged from -2.2429 to 3.7464, with most estimates being positive (83%). The estimated average log odds ratio based on the random-effects model was 1.426 (95% CI -1.157 to 4.008). The outcome did not differ significantly from zero (z = 1.082, *p* = 0.279). A 95% prediction interval for the true outcomes is given by -2.4403 to 4.2793. Therefore, although the average outcome was estimated to be positive, in some studies, the true outcome may be negative. An examination of the studentized residuals revealed that one study (NCT05087381)^33^ had a value larger than ± 2.5758 and maybe a potential outlier in the context of this model. According to the Cook's distances, none of the studies were overly influential. Neither the rank correlation nor the regression test indicated any funnel plot (Fig. 5B) asymmetry *p* = 0.4833 and *p* = 0.5243, respectively).

The doses could be further sub-grouped into studies that used a dose of up to 100 mg/ day, up to 200 mg/day, and up to 300 mg/day (Fig. 6A). There was an apparent difference in effect size correlating with the increase in dose. Up to 100 mg/day had an effect size of 1.43 (95% CI -1.16 to 4.01), which did not differ significantly from zero (z = 1.082, *p* = 0.279). Up to 200 mg/day had an effect size of 0.550 (95% CI 0.236 to 0.865) which differed significantly from zero (z = 3.433 *p* < 0.001), and up to 300 mg/day had an effect size of 1.153 (95% CI 0.593 to 1.713) which differed significantly from zero (z = 4.035 *p* < 0.001). The test for between-subgroup homogeneity resulted in a Q value of 3.592 *p* = 0.166, suggesting that the groups' effect sizes differ.

### Fluvoxamine high/low dose subgroup analysis in COVID-19 mortality

Ten studies included mortality from COVID-19 as an outcome of their study in each subgroup (high with six studies and low with four). However, only five of those studies had a death in one of the groups (fluvoxamine or standard care). Thus, only these studies were included in the subgrouping meta-analysis.

**Figure 7:**
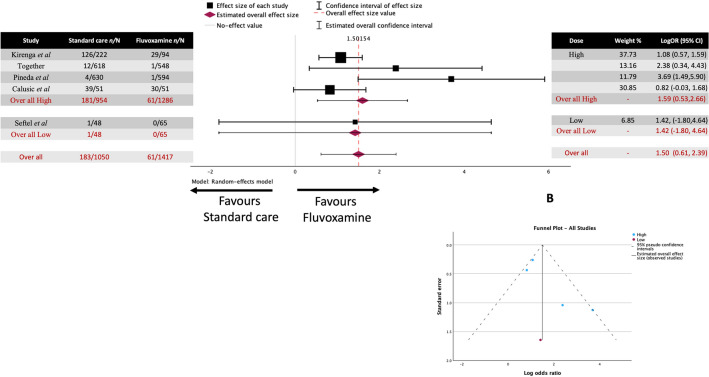
Meta-analysis for all the studies on fluvoxamine treatment of COVID-19 and risk of mortality sub-grouped by dose (High and Low). (**A**) Forrest plot. High dose LogOR 11.593 (95% CI 0.530 to 2.656) z = 2.938, *p* = 0.003. Low dose LogOR 1.420 (95% CI -1.802 to 4.462) z = 0.864, *p* = 0.338. (**B**) Funnel Plots. No Asymmetry was observed in either dose grouping.

The estimated average (Fig. 7) for the high-dose subgroup log odds ratio based on the random-effects model was 1.593 (95% CI 0.530 to 2.656). Therefore, the average outcome differed significantly from zero (z = 2.938, *p* = 0.003).

The estimated average for the low-dose subgroup log odds ratio based on the random-effects model was 1.420 (95% CI -1.802 to 4.462), which did not differ significantly from zero (z = 0.864, *p* = 0.338).

The test between sub-group homogeneity resulted in Q = 0.01, df = 1, and *p* = 0.92, suggesting that the two groups showed heterogeneity (i.e., they were different).

### Fluvoxamine time to treatment subgroup analysis in COVID-19 clinical deterioration

**Figure 8:**
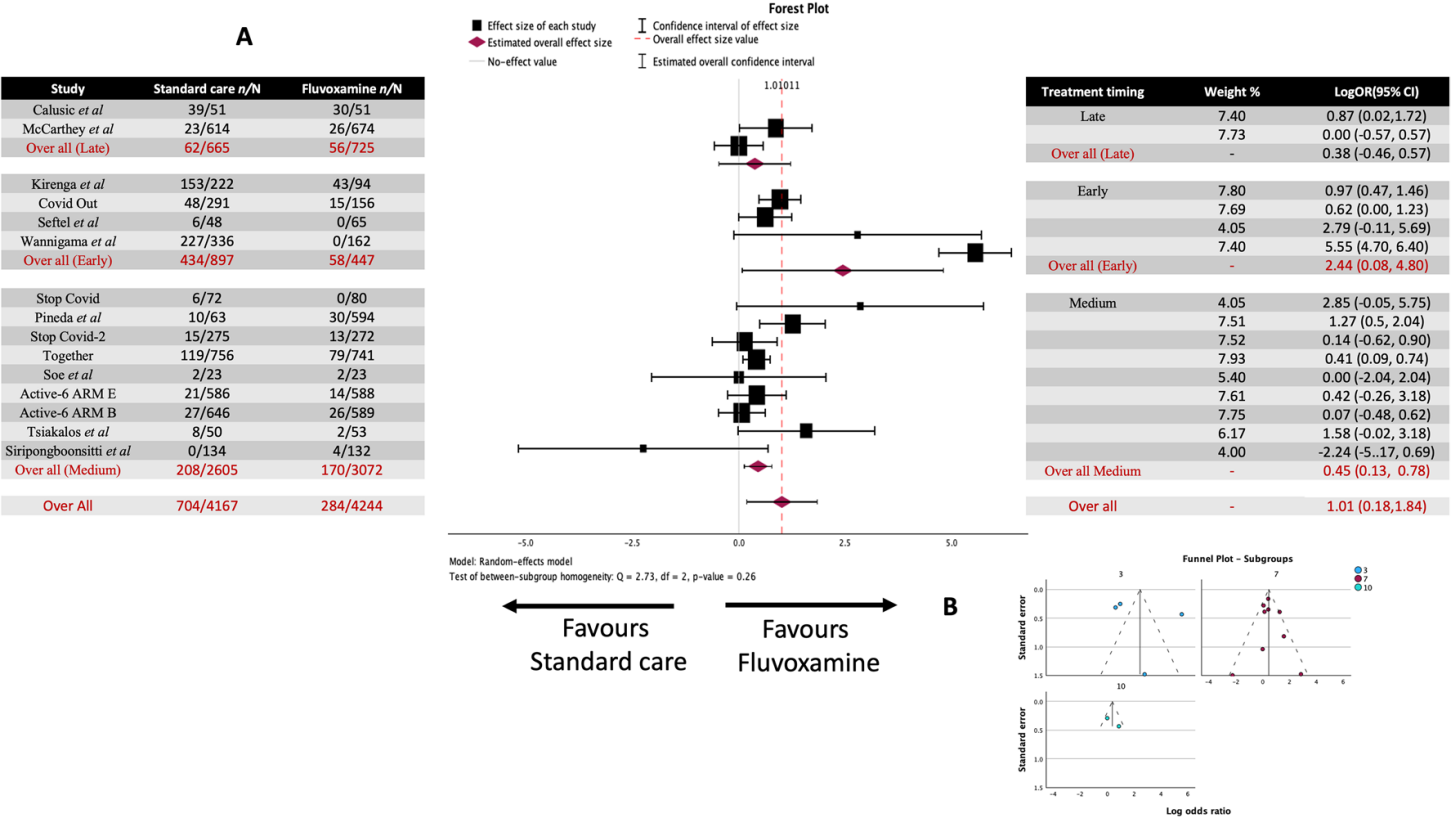
Meta-analysis for all the studies on fluvoxamine treatment of COVID-19 clinical deterioration sub-grouped by time to treatment (early, medium, or late). (**A**) Forrest plots. Early treatment LogOR 2.441 (95% CI 0.078 to 4.805) z = 2.025, *p* = 0.043. Medium treatment LogOR 0.453 (95% CI 0.130 to 0.776) z = 2.748, *p* = 0.006. Late treatment LogOR 0.869 (95% CI 0.09 to 1.718) z = 2.004, *p* = 0.045 (**B**) funnel Plots. No asymmetry was observed at any time point in grouping.

There was one study that started treatment late (up to 10 days), eight studies that began at a medium time point (within up to 7 days), and four studies that started treatment early (up to 3 days). The late subgroup log odds ratio based on the random-effects model was 0.869 (95% CI 0.09 to 1.718) (Fig. 8). The estimated average for the medium time to treatment subgroup log odds ratio based on the random-effects model was 0.453 (95% CI 0.130 to 0.776), which differed significantly from zero (z = 2.748, *p* = 0.006). The estimated average for the early time to treatment subgroup log odds ratio based on the random-effects model was 2.441 (95% CI 0.078 to 4.805. Therefore, the average outcome differed significantly from zero, indicating a potential benefit from fluvoxamine over placebo/standard care (z = 2.025, *p* = 0.043). The test between sub-group homogeneity resulted in Q = 2.730, df = 2, and *p* = 0.255, suggesting that the three groups showed heterogeneity (i.e., they were different). Furthermore, the effect size appears to increase the earlier treatment was started.

### Fluvoxamine time to treatment subgroup analysis in COVID-19 mortality

**Figure 9:**
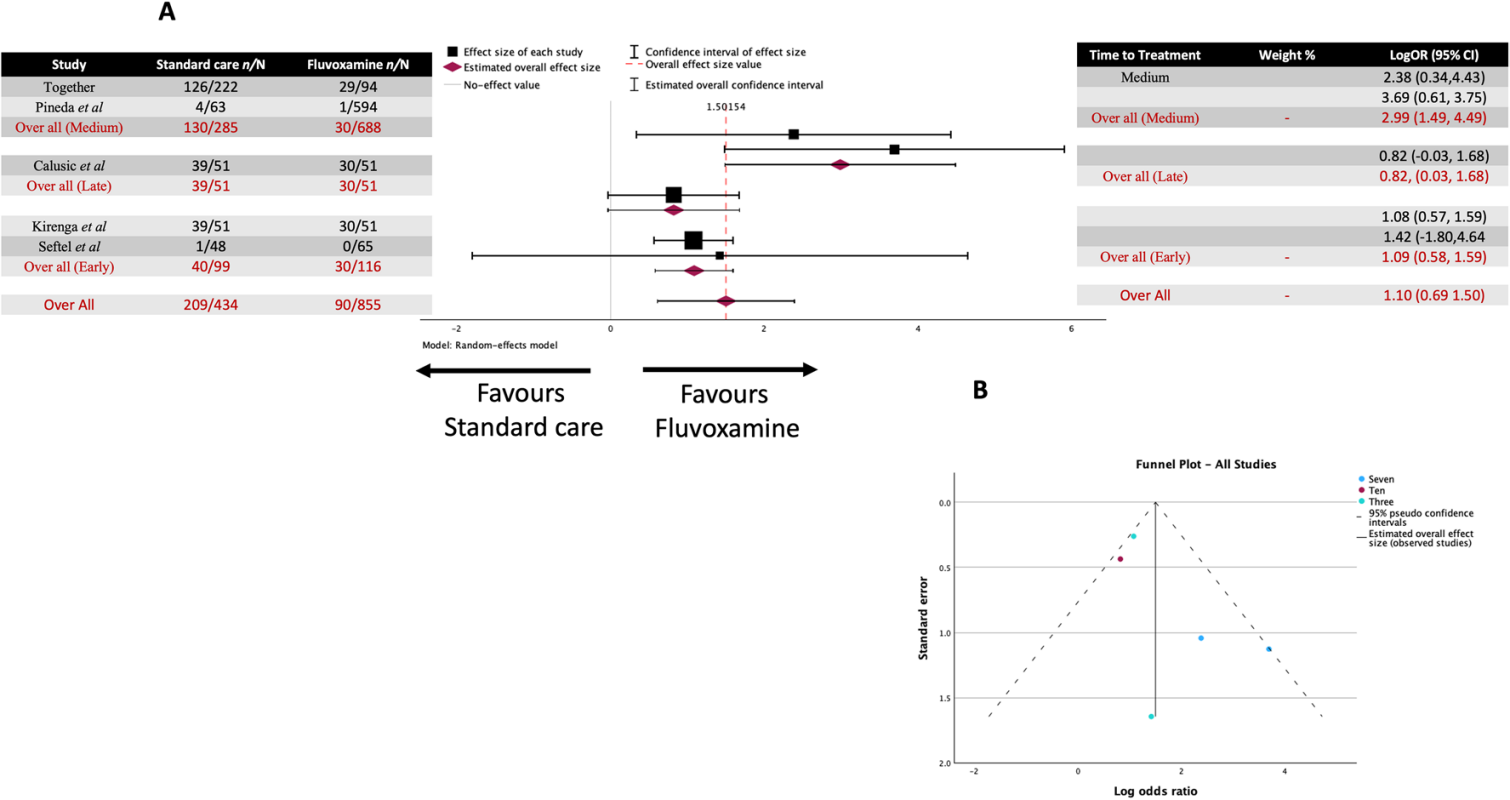
Meta-analysis for fluvoxamine sub-grouped by time to treatment (early, medium, or late) in studies reporting COVID-19 mortality as an endpoint. (**A**) Forrest Plot B Funnel plots. (**B**) Funnel Plot. No Asymmetry was observed at time point grouping.

Ten studies used mortality as a study endpoint regarding fluvoxamine treatment of COVID-19 infection (Fig. 9). Four studies were treated within three days (early), although only two had deaths in one of the groups. Seven studies were treated within seven days (medium), although only two of the studies recorded a death in one of the groups. Finally, two were treated within ten days (late), with only one of these studies recording a death in one of the groups. The effect size was largest in the medium group, 2.988 (95% CI 1.488 to 4.487), which significantly differed from zero, suggesting that fluvoxamine is beneficial compared to placebo/standard care (Z = 3.905, *p* < 0.001), followed by the early group 1.0857 (95% CI 0.582 to 1.593) and differed significantly from zero (Z = 4.216 *p* < 0.001). The lowest effect size was seen in the late group, 0.822 (95% CI -0.032 to 1.676), and this did not differ significantly from zero (Z = 1.886 *p* = 0.059). The test for homogeneity between subgroups resulted in Q = 6.384 (df = 2) (*p* = 0.04).

## Discussion

During the COVID-19 pandemic, hospital bed shortages, particularly in low- and middle-income countries, and stressed healthcare systems, had lead to direct COVID-19 deaths and indirect deaths from untreated conditions^49^. Drug repurposing was aimed to reduce hospitalizations and alleviate this stress, however the time to recovery metric, often used in studies, proved problematic due to variable symptoms and subjective recovery definitions^50^. Instead, clinical deterioration (e.g., hospitalization post-diagnosis) was a better drug effectiveness indicator. Death rates, while examined, were influenced by vaccination uptake, changing disease nature, and emerging variants. Studies with no deaths were excluded to avoid statistical bias from the Haldane-Anscombe correction.

While this is not the first meta-analysis to study the efficacy of fluvoxamine, this study includes more clinical trials and more recent trials than the previous meta-analysis studies. Furthermore, this is the first meta-analysis study to investigate dose and timing as variables for subgrouping, thereby identifying the dose and how soon the intervention was started, significantly affecting the study's outcomes.

In the early days of SARS-CoV-2 infection, the virus could adopt various strategies, such as immune evasion protein expression and epigenetic changes, to delay or fully inhibit host type I interferon (IFN) antiviral defenses. This can lead to pulmonary hyper-inflammation, followed by effects on the heart, kidneys, and other organs. Hospitalization is most probable during this phase at the beginning of the second week of infection^51–54^. This also results in vascular inflammation and damage at both the macro- and microvascular levels, linked to several cardiovascular, metabolic, and neurodegenerative conditions considered part of Long-covid^55^. Curtailing the excessive production of these cytokines early on with the use of fluvoxamine in those clinical trials may have further reduced the risk of clinical deterioration and poor outcomes. Therefore, early treatment appears to be a prerequisite for successful fluvoxamine treatment. Our subgrouping analysis seems to confirm this result. Furthermore, there appears to be an indication of synergy between time to treatment and dose, mainly seen in the subgrouping of the three doses concerning mortality. The higher doses in the Calusic et al*.*^28^ study also correspond to the late treatment. This might explain why the higher effect size of the 300 mg/day treatment did not achieve statistical significance while the lower doses did. However, care needs to be taken when interpreting subgrouping analysis with metadata.

Only one study resulted in a negative logOR^34^. This study compared favipiravir (as a standard treatment) to favipiravir plus fluvoxamine (50 mg two times per day), starting up to four days post-COVID-19 diagnosis. This study used a low dose of fluvoxamine and was categorized as a mid-timepoint treatment. It was one of the most recent studies included in this meta-analysis and was carried out on mild COVID patients. Thus, it was likely a much newer variant of COVID-19 with a lower risk of clinical deterioration. Many studies have proven that the antiviral, favipiravir, offers no benefits in viral clearance or preventing clinical decline^56,57^. Therefore, the rationale for combining fluvoxamine and favipiravir dosing remains unclear and may complicate the interpretation of the results. While the study assesses the prevention of disease progression on the fifth day, it may not be sufficient to understand the sustained effects of fluvoxamine when given in a low dose on the fourth day after COVID-19 diagnosis in a relatively small sample. However, the study showed some benefits of fluvoxamine dose on inflammatory markers, as well as symptom relief, low hospitalization rates, reduced need for oxygen supplementation, decreased intensive care requirements, and zero mortality.

These differences in effects and effect sizes could be related to the mechanism of action via the σ1R and the receptor's expression in the affected tissues at the time of infection. The replication of the coronavirus is associated with the endoplasmic reticulum (ER) and the mitochondria, causing ER stress, unfolded protein response, and mitochondrial depolarization with the release of mitochondrial DNA into the cytoplasm along with other mitochondrial components such as cytochrome-C and calcium ions, which leads to apoptosis and cell death^58–64^. Fluvoxamine has demonstrated the ability to hinder ER/mitochondrial stress and cytokine secretion without impeding traditional inflammatory pathways. This cytoprotective effect may play a role in averting cardiac damage caused by the cytokine storm syndrome triggered by SARS-CoV-2 infection^65–69^. Thus, early treatment with fluvoxamine may prevent, via σ1R activation, the cell damage and cytokine release that leads to clinical deterioration. In contrast, in later treatment, the damage would have already begun at a cellular level, and the fluvoxamine could not prevent the damage and cytokine release, leading to clinical deterioration.

Furthermore, the σ1R is important in virus replication at the early stage of virus infection^70^, as knocking out (KO) or downregulating σ1R resulted in significant decreases in SARS-CoV-2 replication^70^ and σ1R could potentially disrupt the initial phases of host cell reprogramming induced by the virus^71^. All of which make the σ1R an interesting target of fluvoxamine for treating COVID-19. It has also been suggested that fluvoxamine's action against SARS-CoV-2 might be mediated by the σ1R activation increasing endothelial nitric oxide synthase. Fluvoxamine promotes specific eNOS/NO-, AMPK-, and Nrf2/HO-1-mediated defense mechanisms against virus entry and spread^72^.

MicroRNA-155 plays a crucial role in determining the outcome of SARS-CoV-2 infection, which could explain the temporal effects of fluvoxamine. During inflammation, the downregulation of drug-metabolizing cytochromes P450 by miR-155 and other microRNAs may alter fluvoxamine concentrations^73–75^. CYP1A2, CYP2D6, and CYP2C19 metabolize fluvoxamine and its therapeutic concentrations could be significantly impacted by varying miR-155 levels, which differ between young, healthy individuals and older individuals with co-morbidities in COVID-19. This effect depends on the timing, dosage, and recipient of the medication^73,74^.

Another σ1R agonist, citalopram downregulates miR-155 and upregulates SIRT1 expression^76^. Given that fluvoxamine is a more potent S1R agonist than citalopram (Ki = 17.0 nM vs. Ki = 403.8 nM)^77^, it is expected to have a more substantial effect on miR-155. This could explain why very high doses of fluvoxamine (300 mg) might be ineffective, as they could excessively lower miR-155 in patients with already low levels, negatively impacting prognosis. However, there is no published research on the interactions between fluvoxamine and miR-155.

Concerns have been raised that fluvoxamine studies do not demonstrate efficacy, with some even asserting there is "rather explicit evidence of no (relevant) benefit"^78^. However, this systematic review and meta-analysis reveal that, when considering all available studies (placebo-controlled and open-label), fluvoxamine benefits patients by preventing clinical deterioration and death. Even when analyzing only placebo-controlled studies, fluvoxamine is beneficial in avoiding clinical deterioration (death was not studied in enough placebo-controlled trials for a conclusive analysis). Additionally, the review highlights the differences between high and low doses and early versus late treatment.

We acknowledge that many studies in this review were open-label, lacking the rigor of randomized placebo-controlled trials. However, these open-label studies provide valuable real-world evidence of fluvoxamine's effectiveness in mitigating COVID-19 clinical deterioration and mortality, often involving larger sample sizes than placebo-controlled trials. Despite this limitation, our meta-analysis, which excludes non-placebo-controlled trials, still demonstrates the benefit of fluvoxamine in preventing clinical deterioration. It is important to note that there are only seven placebo-controlled studies, which are relatively small compared to some open-label studies.

The COVID OUT study concluded that fluvoxamine (along with other drugs tested) showed no benefit in treating COVID-19. However, the study reported results as a composite of fluvoxamine alone and in combination with Metformin or Ivermectin. Several studies have investigated fluvoxamine in combination with other drugs^23,33,34,79^. While this analysis has focused on the use of fluvoxamine alone, there has been some success with the use of fluvoxamine along with inhaled budesonide^79^, bromhexine, cyproheptadine^33^. However, fluvoxamine in combination with drugs such as favipiravir, metformin, or ivermectin has not been successful^23,34^. The number of combinations of medications trialed with fluvoxamine deserves study per se but is beyond the scope of this systematic review and meta-analysis.

Five studies examined the long-term effects of fluvoxamine in preventing Long-covid, with three already having published data. Overall, these trials indicated a lack of success. However, the COVID OUT trial, which included this follow-up as a post hoc addition, used a "low" dose of fluvoxamine and did not demonstrate efficacy in the initial treatment of COVID-19. Therefore, this result should be interpreted cautiously, considering the numerous studies that did show the clinical efficacy of fluvoxamine. It would be intriguing to see follow-up studies on these findings. For instance, one study focused on investigating fluvoxamine's role in preventing neuropsychiatric symptoms of post-COVID syndrome. It found that fatigue was significantly lower in the fluvoxamine group, along with an expected decrease in depression symptoms^42^. This study had a relatively small sample size, was carried out on vaccinated patients, and used a dose we have subsequently determined as low. This study, while only preliminary and with some limitations, did suggest that fluvoxamine treatment could reduce neuropsychiatric symptoms of Long-covid, although more and more extensive studies are required. Patients undergoing treatment for depression with SSRIs or σ1R activity showed a reduction in risk of developing Long-covid, indicating that SSRIs such as fluvoxamine may have prophylactic or therapeutic effects for Long-covid^43^.

The STOP COVID trials^12,27^ collected follow-up data regarding the degree of recovery toward baseline and persistent COVID-19-related symptoms several months after the acute COVID-19 clinical trial periods. These results were not yet published at the time of the current review, but information on degree of post-acute recovery has now been published^40^. We contacted the authors of these studies (one of whom is an author of this manuscript (AMR)), who suggested that the preliminary analysis for these studies might yield promising results. Those who took fluvoxamine during the acute COVID-19 trial had reduced likelihood of reporting less than 60% recovery back to baseline health. Individual symptom-level Long-covid data from both trials are still undergoing analysis. Further studies are needed with fluvoxamine to confirm whether fluvoxamine could be used as a treatment or whether preloading with fluvoxamine before infection is required to prevent Long-covid effects.

## Conclusions

In this systematic review and meta-analysis, we have identified that fluvoxamine has shown some potential for treating COVID-19 infection, preventing clinical deterioration and mortality. There also appears to be an optimal timing and dose, with early treatment and higher doses being optimal. It is also apparent that the timing of the treatment may be more critical than the dose. The results of this study suggest that treatment within three days of infection, with a dose of 200 mg/ day (or higher if tolerable), is optimal for COVID-19 treatment in the prevention of clinical deterioration and mortality. Furthermore, there is the potential for the prevention of Long-covid symptoms in those who were initially treated for COVID-19 with fluvoxamine. Further study on this is required.

### Supplementary Information

Supplementary Figure 1.

Supplementary Table 1.

## Data Availability

The datasets generated during and/or analysed during the current study are available from the corresponding author on reasonable request.
