# Author Correction: A systematic review and meta-analysis, investigating dose and time of fluvoxamine treatment efficacy for COVID-19 clinical deterioration, death, and Long-COVID complications

**DOI:** 10.1038/s41598-024-67936-4

**Published:** 2024-07-22

**Authors:** Mani Iyer Prasanth, Dhammika Leshan Wannigama, Angela Michelle Reiersen, Premrutai Thitilertdecha, Anchalee Prasansuklab, Tewin Tencomnao, Sirikalaya Brimson, James Michael Brimson

**Affiliations:** 1 Natural Products for Neuroprotection and Anti‑Ageing (Neur‑Age Natura) Research Unit, Chulalongkorn University, Bangkok, 10330 Thailand; 2 Department of Clinical Chemistry, Faculty of Allied Health Sciences, Chulalongkorn University, Bangkok, Thailand; 3 Department of Infectious Diseases and Infection Control, Yamagata Prefectural Central Hospital, Yamagata, Japan; 4 Department of Microbiology, Faculty of Medicine, King Chulalongkorn Memorial Hospital, Chulalongkorn University, Thai Red Cross Society, Bangkok, Thailand; 5 Yamagata Prefectural University of Health Sciences, Kamiyanagi, Yamagata, 990‑2212 Japan; 6 Pathogen Hunter’s Research Collaborative Team, Department of Infectious Diseases and Infection Control, Yamagata Prefectural Central Hospital, Yamagata, Japan; 7 Department of Psychiatry, School of Medicine, Washington University in St. Louis, St. Louis, MO USA; 8 Siriraj Research Group in Immunobiology and Therapeutic Sciences, Faculty of Medicine Siriraj Hospital, Mahidol University, Bangkok, Thailand; 9 College of Public Health Sciences, Chulalongkorn University, Bangkok, Thailand; 10 Department of Clinical Microscopy, Faculty of Allied Health Sciences, Chulalongkorn University, Bangkok, Thailand; 11 Research, Innovation and International Affairs, Faculty of Allied Health Sciences, Chulalongkorn University, 154 Rama 1 Road, Pathumwan, Wang Mai, Bangkok, 10330 Thailand

Correction to: *Scientific Reports* 10.1038/s41598-024-64260-9, published online 12 June 2024

The original version of this Article contained errors.

In the Results, under the subheading ‘Qualitative analysis’, ‘Long-covid studies’,

“A retrospective analysis of patients taking SSRIs at the time of infection showed a % reduction (*p* = 0.0004) in the relative risk of Long-covid among patients (n = 1521) receiving an SSRI with σ1R agonism (fluvoxamine, fluoxetine, escitalopram) and a 21% reduction (*p* = 0.005) in the relative risk of Long-covid among patients (n = 1803) taking an SSRI without σ1R agonism (citalopram, paroxetine, sertraline) compared with patients (n = 14,584) not taking an SSRI^43^. However, as pointed out by K. Hashimoto^44^ this study included citalopram as an SSRI with σ1R activity, which other studies have suggested as incorrect as it does not potentiate NGF-induced neurite outgrowth in PC-12 cells^45^.”

now reads:

“A retrospective analysis of patients taking SSRIs at the time of infection showed a 29% reduction (*p* = 0.0004) in the relative risk of Long-covid among patients (n = 1521) receiving an SSRI with σ1R agonism (fluvoxamine, fluoxetine, escitalopram) and a 21% reduction (*p* = 0.005) in the relative risk of Long-covid among patients (n = 1803) taking an SSRI without σ1R agonism (citalopram, paroxetine, sertraline) compared with patients (n = 14,584) not taking an SSRI^43^. As pointed out by K. Hashimoto,^44^ a preprint of this study included citalopram as an SSRI with σ1R activity, which other studies have suggested as incorrect as it does not potentiate NGF-induced neurite outgrowth in PC-12 cells^45^.”

Furthermore, Reference 43 contained an error and was incorrectly given as:

Sidky, H. et al. Assessing the effect of selective serotonin reuptake inhibitors in the prevention of post-acute sequelae of COVID-19. *medRxiv* (2022).

The correct reference is listed below:

Sidky, H. *et al*. Assessing the effect of selective serotonin reuptake inhibitors in the prevention of post-acute sequelae of COVID-19. *Computational and Structural Biotechnology Journal* **24,** 115–125 (2024). 10.1016/j.csbj.2023.12.045

The original Article has been corrected.

