# Supplementary material for: A systematic review and meta-analysis, investigating dose and time of fluvoxamine treatment efficacy for COVID-19 clinical deterioration, death, and Long-COVID complications
Source: Sci Rep. 2024 Jun 12;14:13462. doi: 10.1038/s41598-024-64260-9 (PMC11166997; doi:10.1038/s41598-024-64260-9)
Supplement: Supplementary file 1 — Supplementary Figure 1. [file 41598_2024_64260_MOESM1_ESM.docx]

**Identification of studies via databases and registers**

Records identified through database

searching (n = 15)

**Identification**

Records screened

(n = 15 )

Records excluded

(n = 0)

**Screening**

Reports assessed for eligibility

(n = 15 )

Reports excluded:

Did not explicitly use fluvoxamine 1 (n = 1)

Studies included in review

(n = 14)

**Included**

Supplementary figure 1: PRISMA Flow diagram for study selection
