# Supplementary material for: A systematic review and meta-analysis, investigating dose and time of fluvoxamine treatment efficacy for COVID-19 clinical deterioration, death, and Long-COVID complications
Source: Sci Rep. 2024 Jun 12;14:13462. doi: 10.1038/s41598-024-64260-9 (PMC11166997; doi:10.1038/s41598-024-64260-9)
Supplement: Supplementary file 2 — Supplementary Table 1. [file 41598_2024_64260_MOESM2_ESM.docx]

Table 1. Quality scoring of literature assessed in this review.

| Question | Stop covid  Lenze et al. | Stop covid-2  Reiersen  et al. | Together  Reis  et al. | Covid Out  Branante  et al. | Activ-6 (ARM B & E). | Soe  et al. | Calusic  et al. | Pineda  et al. | Seftel  et al. | Kirega  et al. | Wannigama  et al. | Siripogboonsitti  et al. | Tsiakalos  et al. | Oskostsky  et al. |
| --- | --- | --- | --- | --- | --- | --- | --- | --- | --- | --- | --- | --- | --- | --- |
| 1 | ✓ | ✓ | ✓ | ✓ | ✓ | ✓ | x | x | x | x | ✓ | ✓ | x | x |
| 2 | ✓ | ✓ | ✓ | ✓ | ✓ | x | - | - | - | - | ✓ | ✓ | - | - |
| 3 | ✓ | ✓ | ✓ | ✓ | ✓ | x | x | x | x | x | x | x | x | x |
| 4 | ✓ | ✓ | ✓ | ✓ | ✓ | ✓ | x | x | x | x | ✓ | ✓ | x | x |
| 5 | ✓ | ✓ | ✓ | ✓ | ✓ | ✓ | ✓ | ✓ | ✓ | ✓ | ✓ | ✓ | ✓ | ✓ |
| 6 | ✓ | ✓ | ✓ | ✓ | ✓ | ✓ | ✓ | ✓ | ✓ | ✓ | ✓ | ✓ | ✓ | ✓ |
| 7 | ✓ | ✓ | ✓ | x | ✓ | ✓ | ✓ | ✓ | ✓ | ✓ | ✓ | x | ✓ | ✓ |
| 8 | ✓ | ✓ | ✓ | ✓ | ✓ | ✓ | ✓ | x | ✓ | ✓ | ✓ | ✓ | x | x |
| 9 | ✓ | ✓ | ✓ | ✓ | ✓ | ✓ | ✓ | ✓ | ✓ | ✓ | ✓ | ✓ | ✓ | ✓ |
| 10 | ✓ | ✓ | ✓ | ✓ | ✓ | ✓ | x | ✓ | ✓ | ✓ | ✓ | x | x | x |
| 11 | ✓ | ✓ | ✓ | ✓ | ✓ | ✓ | ✓ | ✓ | ✓ | ✓ | ✓ | ✓ | ✓ | ✓ |
| 12 | ✓ | ✓ | ✓ | ✓ | ✓ | ✓ | ✓ | ✓ | ✓ | ✓ | ✓ | ✓ | ✓ | ✓ |
| Score | 12 | 12 | 12 | 10 | 12 | 8 | 3 | 3 | 5 | 5 | 10 | 6 | 1 | 1 |

1. Was the study randomized?
2. Was the randomization appropriate?
3. Was the study double-blinded?
4. Was there a description of any withdrawals from the study?
5. Was there a clear description of the inclusion/exclusion criteria for the study?
6. Was there an appropriate control group?
7. Was the dose used appropriate?
8. Were adverse effects monitored and described?
9. Was the method of statistical analysis described?
10. Was there appropriate follow-up of patients?
11. Are the primary and secondary outcomes clearly defined?
12. Have the results of the study been published?
